# Efficacy and safety of supplementation of Exclzyme Pet for the management of arthritis and inflammatory symptoms in dogs: a randomized, double-blind, placebo-controlled pilot trial

**DOI:** 10.3389/fvets.2026.1803915

**Published:** 2026-05-29

**Authors:** Pralhad B. Wangikar, Anand R. Deshpande, Avinash Arja, Kaustubh V. Natu, Ranjeet R. Magdum

**Affiliations:** 1 PRADO, Pune, India; 2 Department of Veterinary Microbiology, COVAS, Parbhani, India; 3 Animedics Health India Pvt. Ltd., Pune, India; 4 Department of Veterinary Surgery and Radiology, Andhra Pradesh Med Tech Zone-Animal Research Centre, Visakhapatnam, India; 5 Department of Veterinary Medicine, Animedics Health India Pvt. Ltd., Pune, India; 6 Department of Veterinary Gynecology, Animedics Health India Pvt. Ltd., Pune, India

**Keywords:** arthritis, Exclzyme Pet, inflammation, lameness, pain, quality of life

## Abstract

**Background:**

A randomized, double-blind, placebo-controlled interventional pilot trial was conducted to evaluate the safety and efficacy of Exclzyme Pet in alleviating clinical symptoms associated with canine arthritis.

**Methods:**

A total of 26 client-owned dogs exhibiting clinical signs of arthritis were enrolled in a 30-day pilot trial, of which 23 completed the study. Subjects were randomly allocated to either the treatment group (*N* = 12), receiving Exclzyme Pet (2 g per dog, administered orally twice daily on an empty stomach with water), or the placebo group (*N* = 11), receiving maltodextrin at an equivalent dose.

**Results:**

Exclzyme Pet resulted in a statistically significant reduction in scores for lameness, weight bearing, joint mobility, willingness to hold up contralateral limb, and pain by 51.96, 46.94, 48.94, 58.0, and 55.56%, respectively and demonstrated clear superiority over placebo. The overall quality of life, mood, and mobility improved significantly in the treatment group over the placebo. Serum C-reactive protein levels decreased markedly by 56.10% in dogs receiving Exclzyme Pet, whereas levels increased by 15.38% in the placebo group, indicating a strong anti-inflammatory effect (*p* < 0.001). No adverse hematological findings or clinical events were reported, and the supplement was well tolerated, without gastrointestinal or systemic complications.

**Conclusion:**

In conclusion, Exclzyme Pet demonstrated significant efficacy in reducing clinical signs of arthritis and improving functional outcomes in dogs, with no evident safety concerns during the entire study period. These results suggest that Exclzyme Pet may serve as a safe and effective adjunctive therapy for managing canine arthritis. Nevertheless, longer-duration clinical trials with large study population are needed to confirm these findings and further elucidate the mechanisms its therapeutic effects.

## 1 Introduction

Arthritis is one of the most prevalent joint disorders encountered in canine practice, often resulting in inflammation, stiffness, and pain, that manifest clinically as a chronic discomfort, progressive loss of joint mobility and function, and lameness. These symptoms collectively diminish the animal’s quality of life and may lead to long-term disability if left unmanaged (1). Beyond developmental or hereditary causes, a range of acquired musculoskeletal conditions contribute to degenerative joint changes. Traumatic injuries can initiate localized cartilage damage that gradually progresses to widespread degeneration. Similarly, ligamentous injuries promote cartilage wear through joint instability, while intra-articular fractures often cause in secondary cartilage loss due to incomplete or abnormal healing (2, 3). Despite its clinical relevance, comprehensive epidemiological data on canine arthritis remain limited, and the prevalence across different breeds and age groups is not yet well established.

Joint destruction in canine arthritis is primarily initiated with the activation of innate immune cells like macrophages and neutrophils, which produces pro-inflammatory cytokines including TNF-α and IL-1β, with activated T lymphocytes also contributing to TNF-α secretion; these early mediators initiate synovial inflammation and set the stage for further damage (4). Subsequently, IL-6 amplifies the inflammatory response and promotes further immune cell recruitment, while IL-17 enhances neutrophil activity that accelerates tissue injury (5). Together, these cytokine-driven processes perpetuate progressive tissue damage, synovial inflammation, and pain observed across various arthritic conditions (6, 7).

Conventional therapeutic management in veterinary practice largely relies on analgesics, non-steroidal anti-inflammatory drugs (NSAIDs), glucocorticoids, and in severe cases, surgical intervention as joint replacement. Weight management is often recommended as an adjunct to reduce mechanical stress on affected joints (6). NSAIDs exert their anti-inflammatory effects through inhibition of cyclooxygenase (COX) enzymes, thereby reducing the synthesis of pro-inflammatory prostaglandin. However, suppression of constitutive COX isoforms may also interfere with physiological homeostasis, predisposing to gastrointestinal, renal, or hepatic adverse effects (8). Given these limitations, increasing attention has been directed toward alternative therapeutic options, including enzyme-based formulations, which may provide anti-inflammatory and analgesic benefits through modulation of inflammatory mediators such as TNF-α, IL-1β, and IL-6, and will support of tissue repair (9). Such biological approaches represent a potentially safer and complementary strategy for the management of both acute and chronic inflammatory joint diseases in companion animals.

Enzyme-based and phyto-therapeutic agents have gained increasing attention as alternative or adjunctive approaches for managing arthritis in veterinary practice. Among these, bromelain, a cysteine protease complex derived from *Ananas comosus* (pineapple), has been associated with a broad spectrum of therapeutic benefits. Beyond its proteolytic function, bromelain contains peroxidase, acid phosphatase, protease inhibitors, and organically bound calcium, maintaining stability across a wide pH range. It’s well documented anti-inflammatory and analgesic properties, in addition to immunomodulatory, and wound healing effects have been supported by both *in-vitro* and *in-vivo* studies, underscoring its multifaceted biological activity (10–12).

Similarly, papain, another proteolytic enzyme derived from *Carica papaya*, has been extensively studied for its potential therapeutic role in managing inflammation in conditions such as arthritis, asthma, and rheumatism (13). Further, serratiopeptidase has also demonstrated promising anti-inflammatory efficacy in acute and chronic inflammatory states. Although its exact mechanism remains incompletely defined, serratiopeptidase is believed to hydrolyze abnormal protein deposits and inflammatory exudates, facilitating their absorption via the circulatory and lymphatic systems. This enzymatic clearance promotes improved local blood flow contributing to pain relief and resolution of inflammation (14). Being a serine protease, serratiopeptidase also exhibits strong affinity for COX-I and COX-II, key regulators within the arachidonic acid cascade responsible for prostaglandins, and thromboxane synthesis (15). Oral administration has been shown to attenuate pain and inflammation by downregulating these pro-inflammatory mediators (16, 17).

Among the phyto-derived agents, Amla (*Phyllanthus emblica*) extract has long been recognized in both traditional and modern medicine for its potent antioxidant and anti-inflammatory properties. Its bioactive constituents—including ascorbic acid, ellagic acid, gallic acid, kaempferol, tannins, and polyphenols contribute synergistically to the neutralization of reactive oxygen species (ROS) and the modulation of inflammatory signaling pathways (18, 19). Additionally, rutin, a flavonoid glycoside of quercetin found in buckwheat, fruits, and vegetables, exhibits a broad pharmacological profile encompassing antioxidant, antimicrobial, and anti-inflammatory effects. Rutin has been shown to inhibit nitric oxide (NO), TNF-α, IL-1β, and IL-6 production as well as LPS-induced NF-κB activation, thereby vascular and systemic inflammatory responses (20, 21).

This randomized, double-blinded, placebo-controlled clinical pilot trial was undertaken to evaluate the safety and efficacy of “Exclzyme Pet,” a proprietary multi-enzyme and phytochemical formulation, in client-owned dogs diagnosed with arthritis. The population considered for the study was consisted of dogs from a diverse range of breeds/crossbreeds. Exclzyme Pet is composed of amylase, bromelain, lipase, papain, Peptizyme SP EN™ (serratiopeptidase) and proteases, and herbal nutraceuticals like amla and rutin. The study aimed to investigate the formulation’s potential in alleviating clinical symptoms associated with inflammatory joint disease. The study was performed at a fixed dose regardless of the body weight of the dogs. Clinical efficacy was systemically assessed through a standardized lameness grading system and pain assessment using the Helsinki Chronic Pain Index (HPCI). Additionally, hematological evaluations and inflammatory biomarkers analyses were performed to monitor systemic responses, assess treatment tolerability, and quantify the overall therapeutic impact of the intervention.

## 2 Material and methods

### 2.1 Materials

#### 2.1.1 Investigational product

The investigational product (IP), Exclzyme Pet, was formulated as a powder, and packaged in 2 g sachets to ensure uniform strength, compliance, and product quality. The placebo formulation consisted solely of maltodextrin. Both, Exclzyme Pet and placebo were supplied by Specialty Enzymes and Probiotics, Chino, USA.

### 2.2 Selection criteria

A total of 26-client-owned adult dogs diagnosed with moderate osteoarthritis were enrolled in this randomized, double blind, placebo-controlled interventional pilot trial. Eligible dogs were aged between 5 and 11 years, of either sex, or exhibited clinical signs of osteoarthritis affecting the hip or elbow joints. Informed consent was obtained from all owners prior to enrolment. A presumptive diagnosis of arthritis was established based on the dog’s medical history, observable clinical signs, and the owner reported concerns. Inclusion criteria further required physical examination and radiographic confirmation of osteophyte formation and subchondral bone sclerosis. To standardize the assessment of lameness, a lameness grading score index was employed and dogs with a score of ≥3 were considered eligible for the study (22).

Dogs were excluded from the study if they met any of the following criteria: (i) Administration of NSAIDs or corticosteroids within 7 days prior to the baseline evaluation; (ii) Presence of clinically significant neurological, systemic, or infectious disease; (iii) Insufficient clinical signs indicative of lameness; (iv) History of surgical intervention on the joint under evaluation; (v) Diagnosis of any musculoskeletal disorder other than osteoarthritis; (vi) Known hypersensitivity to any component of the investigational product; (vii) Pregnancy; or (viii) Recent history of physical trauma. No specific nutritional requirements were mandated; however, owners were advised to maintain their dogs’ routine feeding regimen and avoid major dietary changes throughout the study period.

### 2.3 Study design

The study was approved by The Institutional Animal Ethics Committee (IAEC), Animal Research Centre, AMTZ, Visakhapatnam, India. The experimental procedure received prior approval from the Committee for Control and Supervision of Experiments on Animals (CCSEA), Ministry of Fisheries, Animal Husbandry and Dairying, Government of India, with registration number V-11011(13)/10/2025-CPCSEA-DADF (Date of approval: September 10, 2025). Canine subjects were randomly allocated to either the placebo or Exclzyme Pet treatment group using randomized block design, and the randomization sequence was generated by using simple randomization technique method by the statistician. To ensure integrity, the clinical study was conducted in a double-blinded manner, wherein veterinarians, dog owners, laboratory personnel, and all other staff directly involved in the study were blinded and unaware of the treatment allocation. Blinding was strictly maintained throughout the study and was only broken after completion of the study and finalization of the statistical analysis. Of the 26 enrolled dogs, 23 (mean age = 6.52 ± 0.9 years, mean body weight = 37.88 ± 7.77 kg) successfully completed the 30-day trial, comprising 12 dogs from the treatment group and 11 from the control. Throughout the study, dogs remained under owner care, except during scheduled clinic visits. Physical parameters, including body temperature, body weight, heart rate, and respiratory rate were recorded at baseline and study completion to assess safety and general health.

### 2.4 Treatment protocol

Ready to use sachets of Exclzyme Pet and placebo were provided to the dog owners. Treatments were administered as dietary supplements packed in sachets of uniform size, color, and shape each containing 2 g of product: either placebo (maltodextrin) or Exclzyme Pet. Both products were identical in appearance and distinguished only by unique alphanumeric codes to maintain blinding. Maltodextrin, used as the placebo, is an inert, commonly used excipient that is generally well-tolerated and have no known side effects on joint health or osteoarthritis. Its primary role was to match the physical appearance, texture, and palatability of the investigational product, thereby maintaining blinding of the study. In addition, maltodextrin does contribute some caloric content, but the quantity used (2 g) in the placebo was minimal and unlikely to affect the overall nutritional intake of the dogs. The assigned product (Exclzyme Pet or placebo) was mixed with water, and administered orally twice daily on an empty stomach. Dog owners were instructed to ensure full consumption of each dose and to closely observe and report any adverse reactions or deviations from the dosing schedule throughout the study period.

### 2.5 Efficacy evaluation

The therapeutic efficacy of the treatment was evaluated based on changes in mean lameness grades, and chronic pain indices. This was performed by the attending veterinarians on Day 0 and Day 30. To minimize inter-observer variability, it was made sure that the same veterinarian evaluated respective dog consistently throughout the study.

#### 2.5.1 Lameness grading

The lameness grading system described by Vasseur et al. (22) (Table 1) was utilized as a clinical evaluation tool to assess therapeutic response in dogs affected with osteoarthritis. This system incorporates clinical parameters, including lameness severity, weight-bearing ability, joint mobility, willingness to hold up the contralateral limb, and observable signs of pain in the affected joint (hip or elbow). All assessments were performed by a licensed veterinarian on Day 0 (SOT) and Day 30 (EOT) to monitor improvements in the clinical status.

**Table 1:** Lameness grading system used in the study [reproduced from Vasseur et al. (22)].

Score	Score clinical signs
Lameness	
1	Stands and walks normally
2	Stands normally; slight lameness when walking
3	Stands normally; severe lameness when walking
4	Abnormal posture when standing; severe lameness when walking
5	Reluctant to rise and will not walk more than 5 strides
Weight bearing	
1	Normal weight bearing on all limbs at rest and when walking
2	Normal weight bearing at rest; favors affected limb when walking
3	Partial weight bearing at rest and when walking
4	Partial weight bearing at rest; does not bear weight on affected limb when walking
5	Does not bear weight on limb at rest or when walking
Joint mobility	
1	No limitation of joint movement; no palpable crepitus
2	Mild (10–20%) decrease in range of motion; no palpable crepitus
3	Mild (10–20%) decrease in range of motion with palpable crepitus
4	Moderate (20–50%) decrease in range of motion, palpable joint crepitus
5	Severe (>50%) decrease in range of motion; palpable joint crepitus
Willingness to hold up contralateral limb	
1	Readily accepts contralateral limb being held up and bears full weight on affected limb
2	Offers resistance to elevation of contralateral limb, but bears full weight on affected limb for more than 1 min after contralateral limb is elevated
3	Offers moderate resistance to elevation of contralateral limb and replaces it after 30 s
4	Offers resistance to elevation of contralateral limb and replaces it after 10 s
5	Refuses to raise contralateral limb
Signs of pain	
1	No signs of pain during palpation of affected joint
2	Signs of mild pain during palpation of affected joint; dog turns head in recognition
3	Signs of moderate pain during palpation of affected joint; dog pulls limb away
4	Signs of severe pain during palpation of affected joint; dog localizes or becomes aggressive
5	Dog will not allow examiner to palpate joint

#### 2.5.2 Pain assessment

The treatment response was further assessed using the Helsinki Chronic Pain Index (HCPI-E2) questionnaire (23–25) which was completed by the dog owners. This tool consisted of 11 questions designed to evaluate the severity of chronic pain and its impact on dog’s daily activities and behavior using a numerical scale from 0 to 4. Pain and functional impairment are inherently interconnected in canine osteoarthritis, as ongoing nociceptive input directly affects overall quality of life and mobility patterns. The HCPI assesses key behavioral and functional domains, including mood, willingness to play, frequency of vocalization, ease of movement during walking, trotting, galloping, jumping, lying down, rising, ease of movement after a long rest or major activity. The assessments were done on Day 0 (SOT) and Day 30 (EOT) to capture owner-perceived changes in the dog’s comfort and mobility during the treatment period.

#### 2.5.3 Hematology and inflammatory biomarkers

Blood samples were collected from the cephalic vein of each dog at both the start (SOT) and end (EOT) of the treatment period for the evaluation of hematological and inflammatory biomarkers. The collected samples were analyzed for key parameters, including hematocrit (HCT), hemoglobin (HGB), red blood cell count (RBC), mean corpuscular volume (MCV), mean corpuscular hemoglobin (MCH), mean corpuscular hemoglobin concentration (MCHC), white blood cell count (WBC), granulocyte percentage, lymphocyte percentage, platelet count, and C-reactive protein (CRP) levels. These parameters were assessed to monitor systemic health and to evaluate potential anti-inflammatory effects of the investigational product.

### 2.6 Statistical analysis

All individual data were decoded and compiled for statistical evaluation using Microsoft Excel 2016. Results were expressed as mean ± standard deviation (SD). Data were analyzed at a 95% confidence interval with a significance level of 5% (*p* ≤ 0.05). Intra group (within group) differences between the start (SOT) and end (EOT) of treatment and inter group (between the groups) difference were assessed using were assessed. For continuous data, a t-test was used. The normal distribution of the data was checked using the Kolmogorov–Smirnov Test. Paired and Independent t-tests were used to compare intragroup and intergroup differences in continuous variables, respectively, if the data are normally distributed; otherwise, suitable non-parametric tests-the Wilcoxon signed-rank test and the Mann–Whitney U test were applied, respectively. A *p-*value ≤ 0.05 was considered statistically significant unless otherwise specified.

## 3 Results

A total of 26 client owned dogs diagnosed with osteoarthritis were enrolled in the study, of which 23 dogs (88.5%) successfully completed the trial. The study population included a diverse range of breeds comprising 16 male dogs and 10 female dogs: Labrador Retriever (n = 6), German Shepherd (*n* = 4), Great Dane (*n* = 2), Labrador (*n* = 2), Rottweiler (*n* = 2), Akita (*n* = 1), Alsatian (*n* = 1), Belgian Cross (*n* = 1), Belgian Malinois (*n* = 1), French Bulldog (*n* = 1), Husky (*n* = 1), Indie (*n* = 1), Lab Cross (*n* = 1), Punjab Bully (*n* = 1), and Tibetan Mastiff (*n* = 1). Both the Exclzyme Pet and placebo groups included 13 canine subjects each, randomized equally, and were comparable with respect to sex distribution. None of the animals included in the study had undergone castration or sterilization procedures. The average age of canines was 6.61 ± 1.02 and 6.43 ± 0.78 years, while the average body weight was 37.69 ± 6.51 and 38.08 ± 9.12 kg in the Exclzyme Pet and placebo groups, respectively.

During the course of the study, two dogs died, one due to a snakebite and another from tick fever. Both incidents were deemed to be unrelated with the use of investigational product. Additionally, one subject from the treatment group was withdrawn following a clinical diagnosis of tick fever, which was also determined to be unrelated to the product administration. Furthermore, all the hematology evaluations remained within normal reference ranges throughout the study period, confirming the safety and tolerability of the investigational formulation.

### 3.1 Lameness grading

#### 3.1.1 Lameness

**Figure 1:**
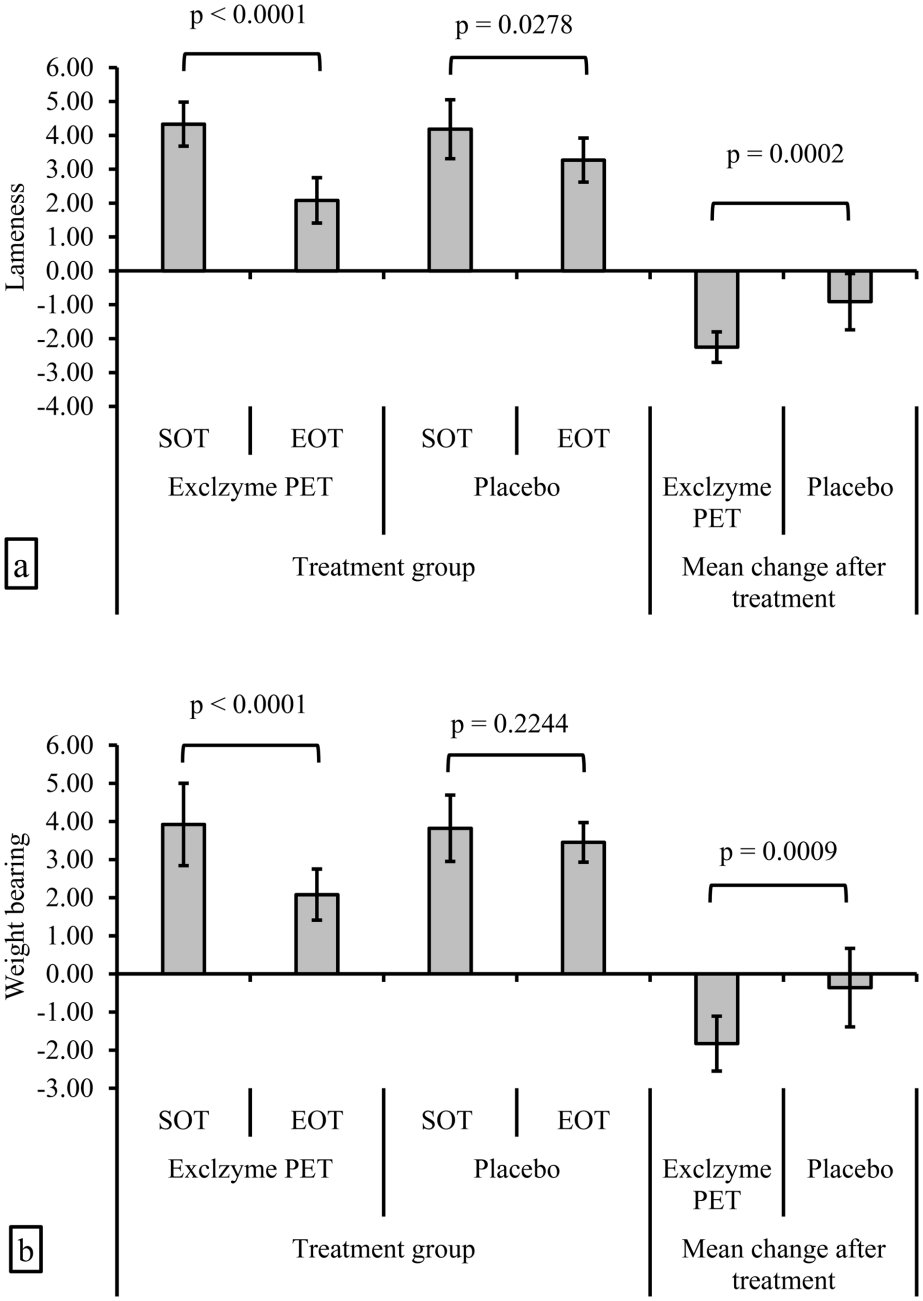
Effect of 30-day oral supplementation of Exclzyme Pet and placebo on **(a)** Lameness and **(b)** Weight bearing in canines with arthritis.

The outcomes of the lameness scoring assessments for all enrolled dogs at SOT and EOT of the trial are presented in Figure 1a. Dogs receiving Exclzyme Pet demonstrated a statistically significant clinical improvement, with mean lameness scores decreasing from 4.33 ± 0.65 (SOT) to 2.08 ± 0.67 (EOT) (*p* < 0.0001). In the placebo group, a reduction in lameness was observed, with scores decreasing from 4.18 ± 0.87 (SOT) to 3.27 ± 0.65 (EOT) (*p* = 0.0278). Although statistically significant, the degree of improvement indicates only a modest clinical benefit. The mean change in lameness scores from SOT to EOT, along with 95% confidence intervals was −2.25 ± 0.45 and [95% CI: −2.54, −1.96] in Exclzyme Pet group, and −0.91 ± 0.83 and [95% CI: −1.47, −0.35] in placebo group, respectively. Intergroup comparison further confirmed that dogs treated with Exclzyme Pet had a 2.48-fold improvement in lameness scores compared with placebo (*p* < 0.0002), underscoring the therapeutic efficacy of the Exclzyme Pet in supporting the reduction of clinical signs of lameness.

#### 3.1.2 Weight bearing capacity

Weight bearing capacity refers to a type of limping or abnormal gait where the dog is able to place some weight on the affected limb, but limb use is noticeably painful or compromised. Assessment of weight-bearing capacity revealed statistically significant improvement in functional limb use among dogs receiving Exclzyme Pet (Figure 1b). In the Exclzyme Pet group, the mean scores decreased from 3.92 ± 1.08 (SOT) to 2.08 ± 0.67 (EOT), with a mean change of −1.83 ± 0.72 [95% CI: −2.29, −1.38], representing a 46.94% reduction (*p* = 0.0009). In contrast, the placebo group showed a statistically non-significant improvement, with scores changing from 3.82 ± 0.87 to 3.45 ± 0.52, corresponding to a mean change of −0.36 ± 1.03 [95% CI: −1.05 to 0.33; *p* = 0.2244]. The intergroup comparison confirmed that the magnitude of change in the Exclzyme Pet group was significantly superior to that observed with placebo (*p* < 0.001). This statistically significant difference demonstrates an efficacy of Exclzyme Pet in assisting the restoration and improvement of weight-bearing functionality in osteoarthritic dogs.

#### 3.1.3 Joint mobility

**Figure 2:**
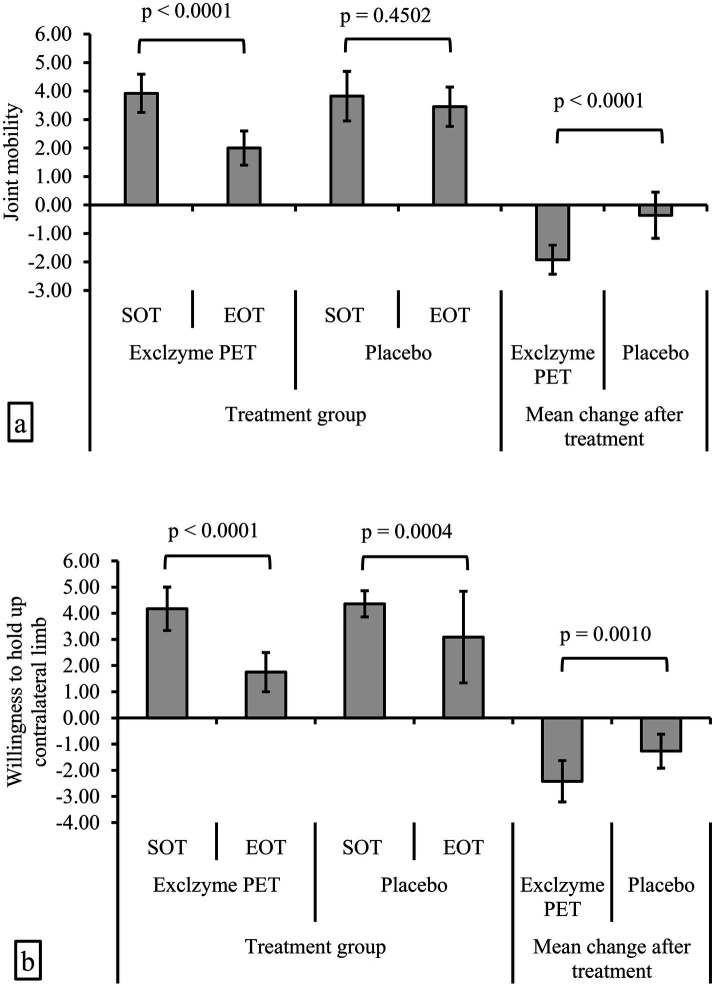
Effect of 30-day oral supplementation of Exclzyme Pet and placebo on **(a)** Joint mobility, and **(b)** Willingness to hold up contralateral limb in canines with arthritis.

Joint mobility reflects a joints ability to move through its normal physiological range of motion. Dogs receiving Exclzyme Pet demonstrated a statistically significant improvement in joint mobility scores over the 30-day treatment period. The mean scores decreased from 3.92 ± 0.67 to 2.00 ± 0.60 at EOT, representing a 48.94% reduction (*p* < 0.0001). In contrast, the placebo group showed a non-significant decline in joint mobility scores, reducing from 3.82 ± 0.87 to 3.45 ± 0.69 (*p* = 0.4502) (Figure 2a). The mean reduction in joint mobility scores with Exclzyme Pet (−1.92 ± 0.51; 95% CI: −2.24, −1.59) was substantially greater than that observed with placebo (−0.36 ± 0.81; 95% CI: −0.91, 0.18). This difference was highly significant (*p* < 0.0001), clearly demonstrating the superior therapeutic effect of Exclzyme Pet in helping the restoration of joint mobility in osteoarthritic dogs.

#### 3.1.4 Willingness to hold contralateral limb

The willingness to hold the contralateral limb during a lameness evaluation is a key clinical indicator, offering insight into the severity of pain, compensatory gait alterations, and the degree of joint inflammation. In this study, dogs receiving Exclzyme Pet showed a statistically considerable enhancement in contralateral limb use. Mean scores decreased from 4.17 ± 0.83 (SOT) to 1.75 ± 0.75 (EOT), corresponding to a mean change of −2.42 ± 0.79 (95% CI: −2.92, −1.91; *p* < 0.0001) (Figure 2b). The placebo group also demonstrated a statistically significant reduction, and the scores decreased from 4.36 ± 0.50 (SOT) to 3.09 ± 0.54 (EOT), with a mean change of −1.27 ± 0.65 (95% CI for mean difference: −1.71, −0.84; *p* = 0.0004). Intergroup comparison confirmed a significantly greater therapeutic effect with Exclzyme Pet (*p* < 0.01), indicating the dogs’ willingness to bear weight on or lift the contralateral limb, indicating a therapeutic benefit in improving limb function.

#### 3.1.5 Pain

**Figure 3:**
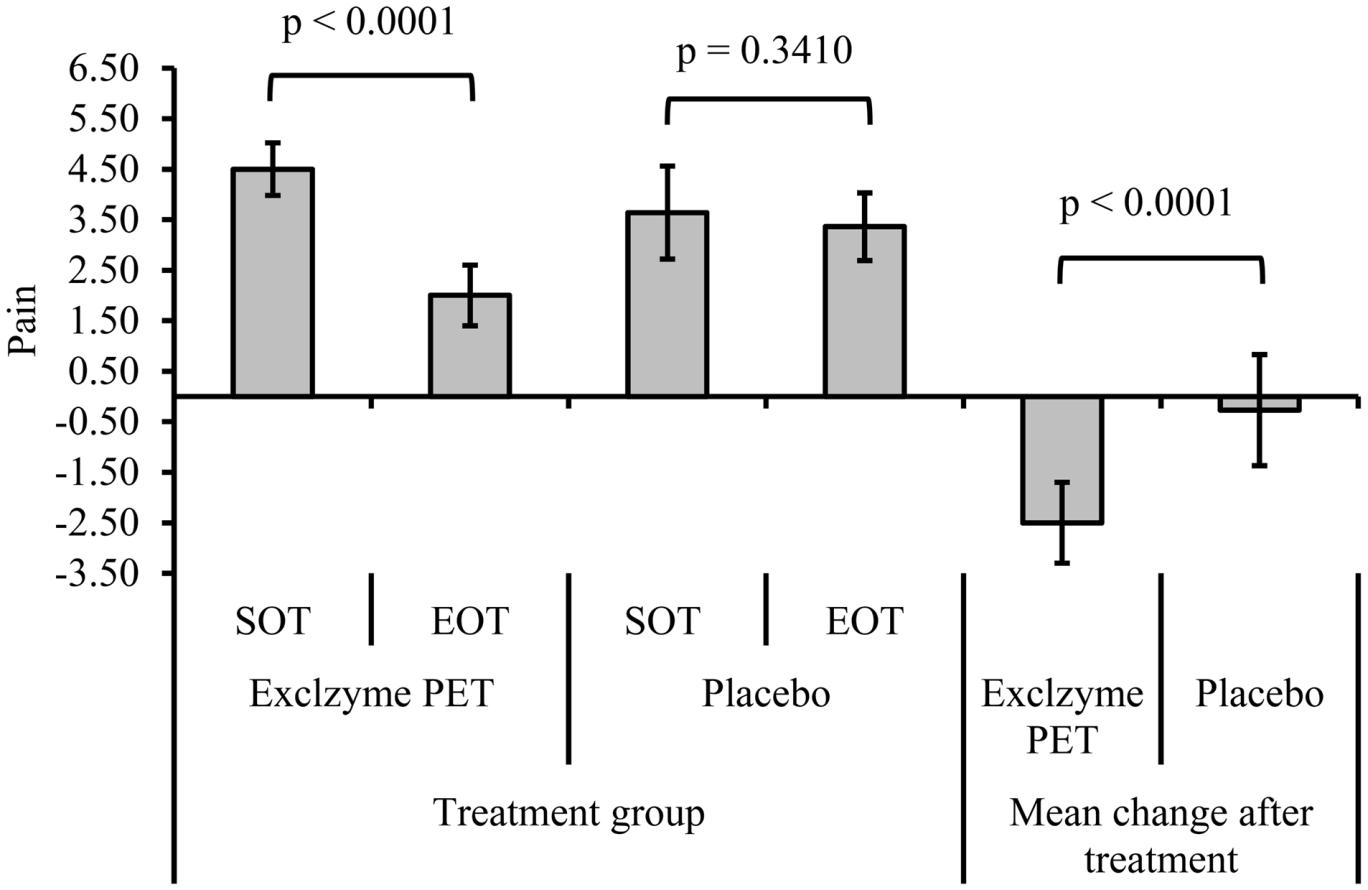
Effect of 30-day oral supplementation of Exclzyme Pet and placebo on pain in canines with arthritis.

Exclzyme Pet demonstrated a statistically substantial reduction in pain scores over 30-day treatment period. The mean scores decreased from 4.50 ± 0.52 at SOT to 2.00 ± 0.60 at EOT (Figure 3), corresponding to a mean decrease of −2.50 ± 0.80 (95% CI: −3.01, −1.99), representing a 55.56% reduction (*p* < 0.0001). In contrast, the placebo group exhibited statistically non-significant reduction in pain, with scores decreasing from 3.64 ± 0.92 to 3.36 ± 0.67, resulting in a mean reduction of −0.27 ± 1.10 [95% CI: −1.01 to 0.47, *p* = 0.3410]. Intergroup comparison confirmed a highly significant treatment effect, with Exclzyme Pet achieving a 9.17-fold greater reduction in pain compared to placebo (*p* < 0.0001). Collectively, these findings demonstrated the efficacy of Exclzyme Pet in assisting the alleviation of clinical signs of pain in osteoarthritic dogs.

### 3.2 Pain assessment

Pain assessment was performed using the Helsinki Chronic Pain Index (HCPI-E2) questionnaire, and the outcomes are presented in Table 2.

**Table 2:** Effect of Exclzyme PET and placebo supplementation on pain assessment parameters in canines with arthritis.

Parameter	Intervention group	SOT	EOT	EOT-SOT	*p*-value (Intra group)	*p*-value (Inter group)	95% CI for mean difference
Dog’s mood	Placebo	2.45 ± 0.69	2.64 ± 0.81	0.18 ± 1.08	0.4502	<0.0001	[−0.54, 0.91]
	Exclzyme PET	2.92 ± 0.79	0.75 ± 0.45	−2.17 ± 0.94	<0.0001		[−2.76, −1.57]
Willingness to play	Placebo	2.82 ± 0.60	2.36 ± 0.50	−0.45 ± 0.82	0.1150	<0.0001	[−1.01, 0.10]
	Exclzyme PET	3 ± 0.74	0.83 ± 0.72	−2.17 ± 1.03	<0.0001		[−2.82, −1.51]
Vocalization frequency	Placebo	2.64 ± 0.50	2.73 ± 0.47	0.09 ± 0.07	0.7180	<0.0001	[−0.38, 0.56]
	Exclzyme PET	3 ± 0.74	1.08 ± 0.67	−1.92 ± 1.00	<0.0001		[−2.55, −1.28]
Ease in walking	Placebo	2.91 ± 0.83	2.36 ± 0.50	−0.55 ± 1.04	0.1396	0.0016	[−1.24, 0.15]
	Exclzyme PET	3.17 ± 0.72	1.08 ± 0.51	−2.08 ± 1.00	<0.0001		[−2.72, −1.45]
Ease in trot	Placebo	3.18 ± 0.75	2.45 ± 0.52	−0.73 ± 0.79	0.0356	<0.0001	[−1.26, −0.20]
	Exclzyme PET	3.33 ± 0.78	0.42 ± 0.51	−2.92 ± 1.00	<0.0001		[−3.55, −2.28]
Ease in gallop	Placebo	2.73 ± 0.79	2.55 ± 0.52	−0.18 ± 1.17	0.6936	0.0003	[−0.97, 0.60]
	Exclzyme PET	2.92 ± 0.67	0.83 ± 0.72	−2.08 ± 0.90	<0.0001		[−2.66, −1.51]
Ease in jump	Placebo	2.73 ± 1.01	2.91 ± 0.30	0.18 ± 0.87	0.6224	0.0039	[−0.41, 0.77]
	Exclzyme PET	2.92 ± 1.00	1.83 ± 0.58	−1.08 ± 1.00	0.0102		[−1.72, −0.45]
Ease in lying	Placebo	2.55 ± 0.52	3 ± 0.63	0.45 ± 0.69	0.1396	<0.0001	[−0.01, 0.92]
	Exclzyme PET	3.50 ± 0.52	2 ± 0.60	−1.50 ± 0.67	0.0003		[−2.22, −0.95]
Rising from lying	Placebo	3.18 ± 0.60	3 ± 0.45	−0.18 ± 0.60	0.5114	<0.0001	[−0.59, 0.22]
	Exclzyme PET	3.50 ± 0.52	2 ± 0.60	−1.50 ± 0.67	0.0001		[−1.93, −1.07]
Ease of movement after long rest	Placebo	2.82 ± 0.87	3 ± 0.45	0.18 ± 0.98	0.7180	0.0043	[−0.48, 0.84]
	Exclzyme PET	3.5 ± 0.52	2.5 ± 0.52	−1.00 ± 0.74	0.0018		[−1.47, −0.53]
Ease of movement after heavy exercise	Placebo	3.09 ± 0.54	3.36 ± 0.50	0.27 ± 0.79	0.3410	0.0110	[−0.26, 0.80]
	Exclzyme PET	3.42 ± 0.51	2.83 ± 0.39	−0.58 ± 0.67	0.0327		[−1.01, −0.16]

#### 3.2.1 Mood

Oral supplementation with Exclzyme Pet resulted in a statistically significant improvement in the dogs’ mood, with scores markedly decreasing from 2.92 ± 0.79 at SOT to 0.75 ± 0.45 at EOT, representing a 74.29% reduction (*p* < 0.0001). This shift reflects a meaningful behavioral transition from “Indifferent” to “Alert,” indicating enhanced emotional responsiveness and overall wellbeing. In contrast, the placebo group exhibited a light increase in mood scores, rising from 2.45 ± 0.69 to 2.64 ± 0.81, suggesting minimal improvement, or a mild decline in affective state. This corresponded to behavioral descriptors ranging from “Neither alert nor indifferent” or “Indifferent.” The mean change in Exclzyme Pet group was −2.17 ± 0.94 [95% CI: −2.76, −1.57], and it was significantly greater than that of a placebo group was 0.18 ± 1.08 [95% CI: −0.54, 0.91]. Intergroup comparison confirmed a highly significant treatment effect (*p* < 0.0001), demonstrating efficacy of the Exclzyme Pet in positively influencing mood and affect in osteoarthritic dogs.

#### 3.2.2 Willingness to play

Exclzyme Pet demonstrated a statistically significant improvement in willingness to play, with scores decreasing by 72.22% from 3.00 ± 0.74 at SOT to 0.83 ± 0.72 at EOT (*p* < 0.0001). This statistically significant reduction reflects a clinically meaningful enhancement in play and overall comfort during routine activities. In contrast, the placebo group showed only a marginal change, with mean scores decreasing from 2.82 ± 0.60 at SOT to 2.36 ± 0.50 by EOT, indicating minimal behavioral response to treatment. The mean change in Exclzyme Pet (2.17 ± 1.03, 95% CI: −2.82, −1.51) corresponds to an improvement from “very reluctantly” to “willing” to play. Conversely, the placebo group demonstrated a mean change of 0.45 ± 0.82 (95% CI: −1.01, 0.10) representing little to no shift, typically remaining between “very reluctantly” to “very reluctantly/sometimes” willing to play. The significant intergroup difference (*p* < 0.001) indicated a therapeutic efficacy, indicating that Exclzyme Pet could help in willingness to play.

#### 3.2.3 Vocalization

Exclzyme Pet demonstrated a considerable reduction in vocalization frequency, with mean scores decreasing from 3.00 ± 0.74 at SOT to 1.08 ± 0.67 at EOT, representing a 63.89% decline (*p* < 0.0001). This reduction in scores reflects a clinically meaningful shift in frequency of vocalization behavior from “often” vocalizing due to discomfort to “hardly ever,” suggesting substantial alleviation of pain-associated distress. In contrast, placebo group showed a slight worsening of the vocalization frequency, with scores increasing from 2.64 ± 0.50 at SOT to 2.73 ± 0.47 at EOT (*p* = 0.7180) indicating no meaningful behavioral improvement. The intergroup analysis revealed a highly significant difference in mean score change by −1.92 ± 1.00 (95% CI: −2.55, −1.28) in Exclzyme Pet group and 0.09 ± 0.07 [95% CI: −0.38 to 0.56; *p* < 0.0001] in placebo group. These results underscore the efficacy of Exclzyme Pet in supporting the reduction of vocalization frequency, a key behavioral indicator of chronic pain and discomfort in dogs.

#### 3.2.4 Walking

Post intervention with Exclzyme Pet, canine subjects exhibited substantially greater improvements in walking over the placebo group. Mean scores decreased from 3.17 ± 0.72 at SOT to 1.08 ± 0.51 at EOT, representing to a statistically significant reduction by 65.79% (*p* < 0.0001). This reflects a clinically meaningful shift in ease of walking—from “with very difficult” at baseline to “with ease” following supplementation. In contrast, the placebo group showed a non-significant reduction, with scores reducing by 18.75% from 2.91 ± 0.83 (SOT) to 2.36 ± 0.50 (EOT) revealing the easiness in walking either remained the same or slightly changed from “with very difficult” to “with difficulty.” The intergroup comparison demonstrated a statically significant difference in mean change scores of by 2.08 ± 1.00 (95% CI: −2.72, −1.45) in Exclzyme Pet group vs. 0.55 ± 1.04 in placebo group (95% CI: −1.24, 0.15) with an overall *p*-value 0.0016. This clearly indicated efficacy of the Exclzyme Pet to help improve the walking mobility in dogs.

#### 3.2.5 Trotting

Both groups demonstrated some degree of improvement in trotting ability following the 30-day intervention period. However, the Exclzyme Pet group exhibited superior outcomes. Mean scores declined from 3.33 ± 0.78 (SOT) to 0.42 ± 0.51 (EOT) representing an 87.50% reduction (*p* < 0.0001). Clinically, this reflected a transition from trotting “with very difficult” at baseline to “with ease” or “with very ease” post supplementation. In comparison, the placebo group showed non-significant reduction, with scores decreasing from 3.18 ± 0.75 to 2.45 ± 0.52 (*p* = 0.0356), indicating that the ease of trotting largely remained unchanged. The intergroup analysis further underscored this difference. The mean change in scores in the Exclzyme Pet group was 2.92 ± 1.00 (95% CI: −3.55, −2.28), whereas the placebo group showed a change of only 0.73 ± 0.79 (95% CI: −1.26, −0.20). The difference between the groups was highly significant (*p* < 0.0001), confirming the superior therapeutic efficacy of Exclzyme Pet in supporting the improvement for ease while trotting.

#### 3.2.6 Galloping

Exclzyme Pet exhibited significant improvement in galloping ability, with mean scores considerably decreasing from 2.92 ± 0.67 (SOT) to 0.83 ± 0.72 (EOT) which corresponded to a 71.43% reduction (*p* < 0.0001). In contrast, the placebo showed a non-significant change, with scores shifting only from 2.73 ± 0.79 at (SOT) to 2.55 ± 0.52 (EOT). The mean change from SOT to EOT was 2.08 ± 0.90 (95% CI: −2.66, −1.51) in the Exclzyme Pet group, compared to 0.18 ± 1.17 (95% CI: −0.97, 0.60) in placebo group. Intergroup comparison confirmed a significant difference (*p* = 0.0003) highlighting the superior functional change in the treatment group. Clinically, dogs receiving Exclzyme Pet improved from galloping “with very difficult” at baseline to “with ease” at the end of treatment, whereas dogs in the placebo group showed virtually no functional change, remaining at “with very difficult.”

#### 3.2.7 Jumping

Dogs supplemented with Exclzyme Pet demonstrated significantly greater improvement in jumping ability compared to the placebo group. The mean change in jumping scores was 1.08 ± 1.00 (95% CI: −1.72, −0.45) for Exclzyme Pet group, which was considerably higher than the placebo group (−0.18 ± 0.87, 95% CI: −0.41, 0.77). This intergroup difference was statistically significant (*p* = 0.0039), confirming the superior efficacy of Exclzyme Pet in supporting the dog’s ease while jumping. Intragroup analysis further demonstrated a 37.14% reduction in the Exclzyme Pet group (*p* = 0.0036), with scores decreasing from 2.92 ± 1.00 (SOT) to 1.83 ± 0.58 (EOT). This reflected a shift in jumping profile from “with very difficult” to “with difficulty.”

#### 3.2.8 Ease in lying down

Following the intervention, Exclzyme Pet group demonstrated a significant improvement in the ease in lying down compared with the placebo group. Scores in the Exclzyme Pet group decreased from 2.92 ± 0.90 (SOT) to 1.33 ± 0.49 (EOT) representing a 54.29% reduction (*p* = 0.0003). This reflected a change lying ability from “with very difficult” to “easily.” In contrast, the placebo group showed a worsening of scores, increasing from 2.55 ± 0.52 (SOT) to 3.00 ± 0.63 (EOT) (*p* = 0.1396). This suggested that the ease of lying either deteriorated slightly or remained “with very difficult.” The mean change in the scores was 1.58 ± 1.00 (95% CI: −2.22, −0.95) for Exclzyme Pet group compared with −0.45 ± 0.69 (95% CI: −0.01, 0.92) in the placebo group. This significant intergroup difference (*p* < 0.0001) confirms the effectiveness of Exclzyme Pet in helping the dogs’ ability to lie down comfortably.

#### 3.2.9 Ease in rising from lying position

The Exclzyme Pet group exhibited a significant improvement in the ease in rising from lying position compared with the placebo group. Mean scores in the Exclzyme Pet group decreased from 3.50 ± 0.52 (SOT) to 2.00 ± 0.60 (EOT), reflecting a decline by 42.86% (*p* = 0.0001). In contrast, the placebo group showed only a non-significant change, with scores changing from 3.18 ± 0.60 at SOT to 3.00 ± 0.45 at EOT (*p* = 0.5114). The mean change in scores was −1.50 ± 0.67 (95% CI: −1.93, −1.07) in the Exclzyme Pet group, compared with 0.18 ± 0.60 (95% CI: −0.59, 0.22) in the placebo group. This highly significant intergroup difference (*p* < 0.0001) highlights the vital role of Exclzyme Pet in supporting smoother and less effortful transitions from lying to standing.

#### 3.2.10 Ease in movement after long rest

In Exclzyme Pet group, the mean mobility scores decreased significantly from 3.50 ± 0.52 at SOT to 2.50 ± 0.52 at EOT (*p* < 0.0001), indicating an improvement from “always difficulty or with difficulty” to “neither easily nor difficultly” in movement after prolonged rest. In contrast, the placebo group showed minimal to no clinical improvement with mean scores changing from 2.82 ± 0.87 (SOT) to 3.00 ± 0.45 (EOT) reflecting a consistent perception of movement to be unchanged, i.e., “with difficulty.” The mean change in scores demonstrated a significant intergroup difference with −1.00 ± 0.74 (95% CI: −0.48, 0.84) in Exclzyme Pet group compared with 0.18 ± 0.98 [95% CI: −1.47 to −0.53; *p* = 0.0043] in placebo group. These findings underscore the role of Exclzyme Pet in aiding mobility following extended period of rest.

#### 3.2.11 Ease of movement after heavy exercise

Following heavy exercise, the Exclzyme Pet group demonstrated an improvement in mobility, with mean scores significantly decreasing from 3.42 ± 0.51 (“always difficulty or with difficulty”) at SOT to 2.83 ± 0.39 (“neither easily nor difficultly”) at EOT (*p* = 0.0327). In contrast, the placebo group showed a mild worsening in mobility, with scores increasing from 3.09 ± 0.54 (“with difficulty”) at SOT to 3.36 ± 0.50 (“with difficulty”) by the EOT. The mean change in scores from was −0.58 ± 0.67 (95% CI: −1.01, −0.16) in Exclzyme Pet group, compared with 0.27 ± 0.79 (95% CI: −0.26, 0.80) in placebo group. Intergroup analysis indicated a significant difference (*p* = 0.0110), providing vital evidence of Exclzyme Pet’s role in supporting recovery and improving mobility after heavy exercise.

The intervention with Exclzyme Pet significantly improved the dog’s clinical signs, as reflected by consistent positive changes across multiple behavioral and mobility parameters. Notably, dogs in the treatment group exhibited a significant improvement in mood, displayed a greater willingness to play, and showcased a reduction in vocalization frequency, indicating enhanced comfort and overall well-being. In addition, the Exclzyme Pet oral supplementation led to a better ease in walking, trotting, galloping, and jumping compared to the placebo group. Improvements were also observed in dogs’ ease in lying down and rising from a lying position, indicating an overall enhancement in mobility. Furthermore, the treatment group demonstrated better mobility after long rests and improved recovery following heavy exercise, collectively highlighting the role of Exclzyme Pet in helping functional movement and reducing discomfort in osteoarthritic dogs.

### 3.3 Safety evaluation: effect on physical and hematological parameters

The effect of oral supplementation of Exclzyme Pet and placebo on the secondary endpoints was evaluated to determine their safety profile in the enrolled canine subjects. Safety assessments included tolerance, adverse events, and serious adverse events, physical as well as hematological parameters. All the side effects occurred during the study were documented by the attending veterinarians, and graded them based on severity. Exclzyme Pet was well tolerated, with no adverse events observed or reported in any dog throughout the study duration. Collectively, these findings indicate that Exclzyme Pet is safe and well tolerated in the studied canine population.

Hematological parameters were evaluated for all canine subjects at both SOT and EOT, and the results are summarized in Table 3. No significant changes were observed in the routine hematological indices across either group. However, C-reactive protein (CRP) levels, a key inflammatory biomarker demonstrated a notable treatment-related response. In Exclzyme Pet group, CRP levels decreased from 0.68 ± 0.22 mg/dL at SOT to 0.30 ± 0.13 mg/dL at EOT, corresponding to a statistically significant 56.10% reduction (*p* = 0.0006, 95% CI: −0.56, −0.21). Conversely, the placebo group, exhibited a mean increase of 15.38% (*p*-value = 0.3246, 95% CI: 0.05, 0.17) with CRP rising from 0.71 ± 0.21 mg/dL to 0.82 ± 0.24 mg/dL. The intergroup comparisons further confirmed a highly significant difference (*p* < 0.0001), indicating anti-inflammatory role of Exclzyme Pet over placebo group.

**Table 3:** Effect of Exclzyme PET and placebo supplementation on hematological parameters in canines with arthritis.

Hematological parameters	Intervention group	SOT	EOT	EOT-SOT	*p*-value (Intra group)	*p*-value (Inter group)	95% CI for mean difference
HCT (%)	Placebo	43.75 ± 5.85	44.40 ± 4.70	−0.65 ± 3.71	0.5767	0.9345	[−1.83, 3.14]
	Exclzyme PET	43.69 ± 7.30	44.15 ± 4.71	−0.46 ± 7.18	0.7075		[−4.10, 5.02]
HGB (g/dL)	Placebo	14.44 ± 1.99	14.85 ± 1.78	−0.42 ± 1.10	0.5114	0.3283	[−0.32, 1.16]
	Exclzyme PET	14.63 ± 2.45	14.41 ± 1.35	0.22 ± 1.86	0.7728		[−1.40, 0.97]
RBC (106/ μL)	Placebo	7.06 ± 1.04	7.25 ± 1.04	−0.18 ± 0.31	0.6458	0.6904	[−0.03, 0.39]
	Exclzyme PET	7.11 ± 1.26	7.23 ± 0.97	−0.12 ± 0.45	0.7950		[−0.17, 0.41]
MCV (fL)	Placebo	66.66 ± 0.56	61.62 ± 17.73	5.05 ± 17.50	0.6458	0.3497	[−16.80, 6.71]
	Exclzyme PET	67.33 ± 0.96	67.46 ± 1.14	−0.13 ± 0.53	0.7508		[−0.21, 0.47]
MCH (pg)	Placebo	22.27 ± 0.66	21.09 ± 4.30	1.18 ± 4.57	0.7676	0.3100	[−4.25, 1.89]
	Exclzyme PET	22.76 ± 0.66	23.05 ± 0.60	−0.29 ± 0.21	0.2040		[0.16, 0.43]
MCHC (g/dL)	Placebo	32 ± 0.73	32.11 ± 0.98	−0.11 ± 0.49	0.9476	0.1908	[−0.22, 0.44]
	Exclzyme PET	32.78 ± 0.75	32.55 ± 1.07	0.23 ± 0.71	0.5426		[−0.68, 0.22]
WBC (10^3^/μL)	Placebo	13.56 ± 2.54	13.39 ± 2.08	0.17 ± 0.88	0.8182	0.1080	[−0.77, 0.42]
	Exclzyme PET	13.58 ± 3.90	12.48 ± 2.86	1.10 ± 1.65	0.3556		[−2.15, −0.05]
Granulocyte (%)	Placebo	67.61 ± 7.73	67.25 ± 7.61	0.35 ± 1.74	0.8955	0.1908	[−1.52, 0.81]
	Exclzyme PET	67.39 ± 10.39	63.88 ± 8.70	3.52 ± 7.69	0.4705		[−8.40, 1.37]
Lymphocyte (%)	Placebo	31.49 ± 7.00	38.84 ± 17.26	−7.35 ± 18.12	0.5114	0.2477	[−4.82, 19.52]
	Exclzyme PET	32.58 ± 10.34	33.18 ± 10.22	−0.60 ± 2.07	0.8625		[−0.71, 1.91]
Platelet (10^3^/μL)	Placebo	407 ± 222.22	358.27 ± 87.62	48.73 ± 176.09	0.9738	0.3492	[−167.03, 69.57]
	Exclzyme PET	386.33 ± 121.49	390.33 ± 111.22	−4.00 ± 27.68	0.8625		[−13.59, 21.59]
CRP (mg/dL)	Placebo	0.71 ± 0.21	0.82 ± 0.24	−0.11 ± 0.09	0.3246	<0.0001	[0.05, 0.17]
	Exclzyme PET	0.68 ± 0.22	0.30 ± 0.13	0.38 ± 0.27	0.0006		[−0.56, −0.21]

All the vital hematological parameters including hematocrit, hemoglobin, red blood cell count, mean cell volume, mean cell hemoglobin, mean cell hemoglobin concentration, white blood cell, granulocytes, lymphocytes, and platelet count, remained well within the normal biological reference ranges throughout the study. This stability indicates that the investigational product did not induce any adverse effects on hematological parameters. The minor fluctuations observed within and between groups at SOT and EOT were statistically non-significant (*p* ≥ 0.05).

## 4 Discussion

This randomized, double blind, placebo-controlled pilot trial was designed to assess the efficacy of Exclzyme Pet in the therapeutic management of canine arthritis. The formulation contains a blend of amylase, bromelain, lipase, papain, Peptizyme SP EN™ (serratiopeptidase) and proteases, and herbal nutraceuticals like amla and rutin. The therapeutic potential of the product was assessed using a structured set of primary outcome measures comprising lameness scoring and pain assessment. Secondary outcome measures included the physical and hematological parameters to determine systemic safety and physiological tolerability. This integrated assessment framework enabled a comprehensive evaluation of the clinical efficacy and safety profile of Exclzyme Pet in the arthritic canine subjects.

The 30-day oral supplementation of Exclzyme Pet resulted in a significant improvement in statistically reducing the scores for lameness and associated clinical parameters. The lameness scores reduced by 51.96% in Exclzyme Pet group, a markedly greater improvement compared to the placebo group (*p* < 0.0002). This reduction in lameness scores reflected a clinical shift from “abnormal posture when standing; severe lameness when walking” to “stands normally; slight lameness when walking.” Exclzyme Pet also produced significant improvements in the key components of the lameness-grading index, including weight bearing ability, joint mobility, willingness to hold up contralateral limb and pain response. Reductions in these parameters scores were statistically significant compared to placebo (*p* < 0.001).

The Helsinki Chronic Pain Index (HCPI-E2) questionnaire is a validated tool which evaluates pain-related behaviors across multiple domains such as mood, mobility, and social interaction, providing a multidimensional understanding of chronic pain. Previous research has demonstrated strong correlations between HCPI scores and veterinary clinical assessments, supporting the reliability of owner-derived data. However, as with any subjective measures, variability in owners’ perception of discomfort and the potential for reporting bias remain inherent limitations. To mitigate these factors, owners received clear, standardized instructions, and repeated assessments were completed by the same individual to promote consistency. Overall, the HCPI proved to be a practical, non-invasive, and sensitive method for monitoring chronic pain in dogs, reinforcing the value of owner insights in evaluating conditions that affect canine quality of life over time. The findings revealed statistically significant and clinically meaningful improvements in both behavioral and physical parameters in the Exclzyme Pet group compared with the placebo group. Notable improvements were observed in trotting (87.50%), mood (74.29%), playfulness (72.22%), vocalization (63.89%), ease of lying down (54.29%), and rising from a lying position (42.86%) with between-group differences at *p* < 0.001, indicating a strong therapeutic impact on affective state and mobility. Additional improvements were identified in galloping (71.43%), walking (65.79%), jumping (37.14%), and movement after rest (28.57%) in the Exclzyme Pet group compared with placebo (*p* < 0.01), along with enhanced post-exercise mobility (17.07%, *p* < 0.05). In contrast, the placebo group demonstrated minimal improvement or slight deterioration across most behavioral and mobility parameters. The consistent statistical significance across multiple domains underscores the effectiveness of Exclzyme Pet in supporting the resolution of pain-related behaviors and improving locomotor function in dogs with chronic pain.

Dogs with inflammatory limb pain often shift weight away from the affected leg, leading to lameness and reduced mechanical stress. This altered behavior results in altered gait, lower activity, and reluctance to jump, reflecting both pain severity and joint dysfunction (26). Inflammation and pain are central drivers in the progression of arthritis, amplifying tissue damage and exacerbating functional impairment. Serratiopeptidase have shown their potential in breakdown of inflammatory mediators, reducing pain and which may contribute in reduction of lameness, improves tissue healing in musculoskeletal conditions. In line with findings by Misraulia et al. serratiopeptidase has shown notable anti-inflammatory efficacy in both acute and chronic conditions. Although its mechanism is not fully defined, it is thought to hydrolyze abnormal protein deposits and inflammatory exudates, facilitating their clearance via circulatory and lymphatic pathways. This enzymatic activity enhances local blood flow and contributes to pain relief and resolution of inflammation (14). Proteases (such as bromelain, papain, and Peptizyme SP EN in Exclzyme Pet) modulate inflammation by mimicking plasmin activity, acting on fibrinogen to enhance fibrinolysis, and promoting anti-inflammatory prostaglandin synthesis. The substrate specificity of proteases is closely parallel to plasmin, thereby supporting both inflammatory control and fibrin breakdown which may contribute to alleviate inflammation and pain (27–29). Beyond their systemic effects, proteases and lipases demonstrate notable gut-protective properties. *In-vitro* evidence indicates that proteases exert antioxidant and anti-inflammatory actions, while lipases downregulate pro-inflammatory lipopolysaccharide-producing genes and enhance the synthesis of anti-inflammatory D-amino acids. Collectively, these enzymes improve intestinal barrier integrity, enrich beneficial microbial populations, and increase short-chain fatty acids (SCFAs), which are critical mediators of systemic anti-inflammatory responses (30–32). Exogenous enzymes are increasingly incorporated into veterinary nutraceutical formulations for their capacity to modulate inflammatory pathways, reduce systemic inflammatory burden, and enhance nutrient bioavailability. In a trial where dogs and cats were fed protease-rich diets, these enzymes demonstrated beneficial effects on gastrointestinal microbiota, contributing to reduced chronic inflammation, observed to be a relevant therapeutic strategy for managing arthritis and other inflammatory conditions in canines (33, 34).

Key bioactive constituents of Exclzyme Pet have demonstrated substantial anti-inflammatory activity both *in-vitro* and *in-vivo* models, supporting their therapeutic relevance in modulating joint inflammation associated with arthritic conditions. Bromelain exerts multimodal anti-inflammatory actions. In a report presented by Livio et al. bromelain demonstrated a notable reduction in plasma fibrinogen levels, thereby helping to regulate clotting and inflammatory responses (35) and supporting suppression of bradykinin a vasoactive peptide strongly implicated in pain and vasodilation; and enhancing serum fibrinolytic activity, facilitating fibrin degradation to reduce swelling and edema (36). In canine chondrocytes, bromelain significantly reduced apoptosis and promoted cell proliferation without compromising cell viability (*p* < 0.05). It also help downregulate the expression of tissue inhibitor of metalloproteinase-1 (TIMP-1) and matrix metalloproteinase-3 (MMP-3) indicating anti-catabolic effects and cartilage protective properties. These findings highlight bromelain’s capacity to support cartilage repair and modulate osteoarthritic pathways. Collectively, it’s fibrinolytic, anti-edematous, and anti-catabolic actions contribute to reduced vascular permeability and effective mitigation of pain and inflammation (11).

Papain also has demonstrated substantial anti-inflammatory activity across multiple experimental models. *In vitro* studies have shown that Papain significantly reduced carrageenan-induced paw edema, cotton pellet-induced granuloma formation, and inflammation in arthritic rat models, underscoring its potent anti-inflammatory activity (13). Similar anti-inflammatory outcomes have been reported with papaya extract in independent studies, further supporting its therapeutic potential (13). Clinical evidence also highlights papain’s efficacy in patients with lumbar spine osteoarthritis, wherein its supplementation led to significant reductions in pain (visual analog scale—VAS, *p* = 0.001) and disability (*p* = 0.000), along with improvements in quality of life. Notable effects were additionally observed in biochemical markers, including alkaline phosphatase ALP (*p* = 0.054) and serum creatinine (*p* = 0.035), indicating systemic benefits alongside symptom relief (37). Several studies suggest that papain exhibits anti-inflammatory activity, comparable to that of conventional NSAIDs, and its mechanism of action is believed to involve pathways similar to those targeted by NSAIDs, positioning papain as a promising natural alternative for managing inflammation-associated conditions.

Serratiopeptidase has consistently demonstrated strong anti-inflammatory and analgesic activity across multiple preclinical models. Its therapeutic effect is primarily attributed to its proteolytic and mucolytic properties, which facilitate the breakdown of inflammatory exudates and abnormal proteins. This degradation promotes enhanced absorption and clearance of inflammatory by-products through the blood and lymphatic systems, thereby contributing to reduced swelling and improved tissue recovery. In a controlled experimental study performed by Jadhav et al. involving healthy canine subjects with turpentine oil-induced acute paw edema, serratiopeptidase produced the greatest reduction in edema volume when compared with standard anti-inflammatory agents such as ibuprofen and nimesulide (15). Additionally, in a similar *in-vitro* study involving rats with inflammation, serratiopeptidase exhibited a significant antinociceptive effect, demonstrating its potential to alleviate pain associated with inflammatory processes (38).

Amla, a natural source of ascorbic acid, polyphenols, and antioxidants, exhibits notable antioxidant and anti-inflammatory and anti-collagenase activity (39). An *in-vitro* study performed by Sumantran et al. using human arthritic cartilage, amla fruits powder has demonstrated a statistically significant, dose dependent inhibition of collagenase type II activity demonstrating short-term chondro-protective effects and suggesting its relatable potential with current outcomes, to slow cartilage degradation in osteoarthritic conditions (40). Amla has also shown a significant reduction of edema and granuloma formation in the rexin pellet method (41) and showed a dose-dependent, statistically significant reduction in paw edema volume in both acute and chronic inflammatory models (*p* < 0.001) (42). These findings position amla as a promising natural alternative to conventional anti-inflammatory therapies. Rutin, a flavonoid, has also demonstrated strong anti-inflammatory activity across various models. It significantly inhibited inflammatory responses in LPS-stimulated RAW 264.7 macrophages (43) and exhibited comparable effects in lipopolysaccharide-stimulated canine macrophage cells (20). In canine studies, rutin administration enhanced lymphatic flow, while *in-vitro* studies demonstrated potent antioxidant activity and cellular morphology protection effect under oxidative stress (44–46). Collectively, these findings highlight rutin’s capacity to modulate inflammatory mediators and enhance cellular resilience, making its effects highly relevant to the present results.

Research indicates that enzyme and herbal blends can play a supportive role in muscle recovery and tissue repair in canines, contributing to improved functional outcomes in inflammatory conditions (46). In a previous investigation by Cardeccia et al. a proprietary formulation containing bromelain and other anti-inflammatory agents demonstrated significant improvement in clinical signs among affected dogs (47). Further, the cannabis and cannabinoids based formulations have demonstrated improvements in pain, seizures, and behavior in dogs with no serious adverse events, and precisely covered in a systemic review (48). Another systematic review and meta-analysis of animal intervention studies indicated positive role of cannabidiol in reducing the pain severity scores and pain interference scores in canines with osteoarthritis (49). But the long-term efficacy, optimal dosages, and safety for medicinal use of cannabis, cannabinoids and cannabidiol remain unclear (48, 49). In the present study, comparable outcomes were observed; further suggesting that such enzyme based formulations may be effective for short-term management of arthritis related discomfort and mobility impairments in canine subjects.

The bioactive components such as bromelain, papain, and other proteolytic enzymes have consistently demonstrated anti-inflammatory, analgesic, and anti-edematous properties in preclinical and clinical models. Their complementary mechanisms suggest a potential synergistic contribution to symptomatic relief in arthritic dogs. In the present study, Exclzyme Pet was well tolerated at the recommended dosage across all enrolled canine subjects, with no treatment-related adverse effects reported and all clinical and physiological parameters remaining within normal limits throughout the intervention period. While these findings support the product’s safety and its preliminary therapeutic potential. The current pilot study was conducted on limited subjects and short study duration, and future research involving larger cohorts, prolonged intervention period, multiple locations, and structured dose-escalation protocols is needed to warrant these preliminary findings. Such studies would help refine its safety margins, clarify dose-relationships, and better establish the long-term efficacy of Exclzyme Pet in the clinical management of canine arthritis.

## 5 Conclusion

This randomized, double blind, placebo-controlled pilot trial for 30-day study demonstrated both the clinical efficacy and safety of Exclzyme Pet in management of canine arthritis. Dogs receiving the intervention exhibited significant reduction in lameness-associated parameters, alongside marked improvements in mobility, pain, weight-bearing, and overall quality of life. These clinical gains were further supported by notable reduction in CRP levels, indicating meaningful anti-inflammatory effects compared to placebo group. Throughout the study period, no adverse events or safety concerns emerged, and all physical and hematological parameters remained within normal physiological limits, underscoring the product’s favorable safety profile. Collectively, these results highlight the therapeutic potential of enzyme-based formulations as viable non-pharmaceutical options in management of chronic musculoskeletal conditions in veterinary practice. Nevertheless, larger scale clinical trials with multiple locations and prolonged durations and mechanistic investigations are warranted to validate these findings, refine dose response relationships, and further elucidate the pathways underlying the observed clinical benefits.

## Data Availability

The raw data supporting the conclusions of this article will be made available by the authors on request.
